# Identification of Celastrol as a Novel YAP-TEAD Inhibitor for Cancer Therapy by High Throughput Screening with Ultrasensitive *YAP/TAZ–TEAD* Biosensors

**DOI:** 10.3390/cancers11101596

**Published:** 2019-10-19

**Authors:** Kazem Nouri, Taha Azad, Min Ling, Helena J. Janse van Rensburg, Alexander Pipchuk, He Shen, Yawei Hao, Jianmin Zhang, Xiaolong Yang

**Affiliations:** 1 Department of Pathology and Molecular Medicine, Queen’s University, Kingston, ON K7L 3N6, Canada; kazem.nouri@queensu.ca (K.N.); tahaazad66@gmail.com (T.A.); min.ling@queensu.ca (M.L.); 8vrhjj@queensu.ca (H.J.J.v.R.); 15ap63@queensu.ca (A.P.); yh5@queensu.ca (Y.H.); 2 Department of Cancer Genetics and Genomics, Roswell Park Cancer Institute, Buffalo, NY 14263, USA; He.Shen@RoswellPark.org (H.S.); jianmin.zhang@roswellpark.org (J.Z.)

**Keywords:** NanoLuc, biosensor, Hippo pathway, high throughput screening, cancer

## Abstract

The Hippo pathway has emerged as a key signaling pathway that regulates a broad range of biological functions, and dysregulation of the Hippo pathway is a feature of a variety of cancers. Given this, some have suggested that disrupting the interaction of the Hippo core component YAP and its paralog TAZ with transcriptional factor TEAD may be an effective strategy for cancer therapy. However, there are currently no clinically available drugs targeting the YAP/TAZ–TEAD interaction for cancer treatment. To facilitate screens for small molecule compounds that disrupt the YAP–TEAD interaction, we have developed the first ultra-bright NanoLuc biosensor to quantify YAP/TAZ–TEAD protein–protein interaction (PPI) both in living cells and also in vitro using biosensor fusion proteins purified from bacteria. Using this biosensor, we have performed an in vitro high throughput screen (HTS) of small molecule compounds and have identified and validated the drug Celastrol as a novel inhibitor of YAP/TAZ–TEAD interaction. We have also demonstrated that Celastrol can inhibit cancer cell proliferation, transformation, and cell migration. In this study, we describe a new inhibitor of the YAP/TAZ–TEAD interaction warranting further investigation and offer a novel biosensor tool for the discovery of other new Hippo-targeting drugs in future work.

## 1. Introduction

The Hippo signaling pathway was originally identified as the key regulator of organ size control [1,2,3,4]. More recent studies have revealed that the Hippo pathway is frequently dysregulated in a variety of cancers [5,6,7]. It represents a major cell signaling network, which negatively regulates the activity of the oncogenic transcriptional coactivator YAP and its paralog TAZ [8,9,10]. The mammalian Hippo pathway is activated in response to various stimuli such as cell–cell contact, nutrient starvation, and DNA damage, which results in a sequential phosphorylation cascade of serine/threonine kinases MST1/2 and LATS1/2 that culminates with the phosphorylation of YAP/TAZ. This phosphorylation prevents YAP/TAZ from translocating to the nucleus, binding to the TEAD family of transcription factors and promoting the expression of key downstream target genes such as CTGF, CYR61, FGF1, and PD-L1. These downstream genes are known to regulate several cancer hallmarks such as cell proliferation, cell motility, angiogenesis, and immune evasion [1,11,12,13,14].

Since TEAD is the major transcription factor mediating YAP/TAZ oncogenic functions, it has been proposed that direct disruption of the YAP/TAZ–TEAD interaction would be an attractive strategy to restrain the transcriptional outputs of YAP/TAZ and the Hippo pathway for cancer therapy [13,15,16,17]. Indeed, several attempts to generate YAP–TEAD inhibitors have been reported in the literature. In 2012, Verteporfin (VP), a photosensitizer used for treating macular degeneration, was identified as the first small molecule inhibitor (SMI) that targets YAP–TEAD in a screen for inhibitors amongst FDA-approved drugs [18]. Subsequent work revealed VP was unlikely to hold value for YAP–TEAD inhibition in cancer patients, as it is difficult to synthesize in large scale, has limited solubility and stability, requires high concentrations for anti-tumor effects, and has Hippo-independent effects [17,19,20]. Other SMIs (e.g., flufenamic acid and CA3) were subsequently reported to regulate YAP–TEAD activity, although neither of these agents has been shown to directly disrupt the YAP/TAZ–TEAD interaction [21,22]. Recently, two therapeutic hot spots at TEAD–YAP interfaces have been identified and used as the structural basis for the design of peptides to antagonize the YAP/TAZ–TEAD interface using structural biology and modeling. These included “Peptide 17” (P17), which did effectively disrupt the YAP–TEAD interaction but is likely to suffer the typical challenges associated with using peptides as therapeutic agents (i.e., low chemical and physical stability, short half-life in plasma) [15,23,24]. Finally, using a similar structural analysis approach, a single small molecule compound (CPD3.1) has been identified to disrupt YAP–TEAD interaction [25]. Despite these efforts, at present time there are no small molecule compounds that have progressed to clinical trials for cancer therapy. To maximize the likelihood of identifying agents that hold therapeutic value, HTS must be performed that yield numerous candidate YAP–TEAD inhibitors with varying pharmacological and phamacokinetic properties.

**Figure 1:**
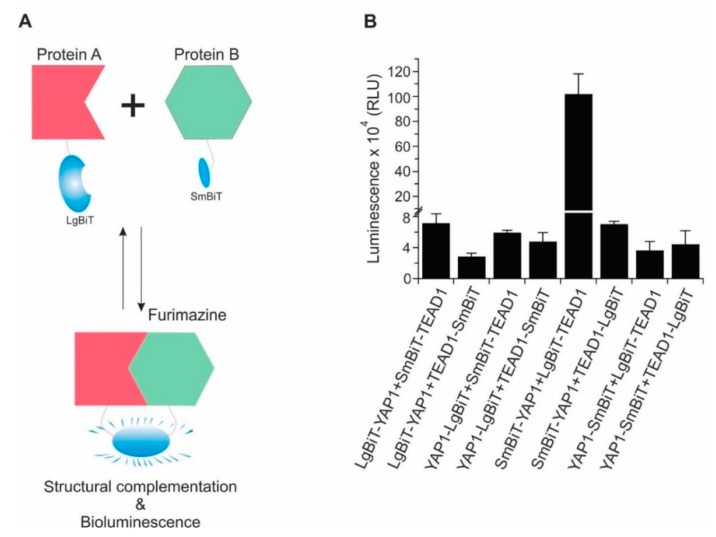
(**A**) Schematic diagram of the NanoBiT protein-protein interaction assay. (**B**) Identification of the best probe pairs for detecting YAP–TEAD interaction using different orientations of YAP and TEAD and SmBiT/LgBiT fragments. Each pair of biosensors were transfected into HEK293T cells. Biosensor activity was determined 48 h after transfection using NanoBiT assay. The order of writing represents the N-terminus or C-terminus orientation of LgBiT and SmBiT (e.g., LgBiT–YAP1 denotes that LgBiT is fused on N-terminus of the YAP1 fragment). Data are represented as mean ± SD (*n* = 3).

Bioluminescence methodologies have proven incredibly useful in the noninvasive monitoring of a wide range of biological activities such as quantitation of cell viability, apoptosis, various processes related to cellular metabolism, gene expression, protein localization, and disease progression [26,27,28,29]. Their high sensitivity, operational simplicity, and wide dynamic range have made them a valuable research tool. Luciferase enzymes have been exploited for the majority of such applications. One of the common applications of luciferase is to circularly permute these proteins to create biosensors capable of monitoring protein-protein interactions (PPIs) using complementary assays [30,31,32]. Conventionally, the firefly and Renilla luciferases (FLuc or RLuc) have been the most widely used luciferases for bioluminescent imaging, although at times, their utility can be limited due to their low stability, large size, and short half-life [33,34]. Recently, a stable, small, and bright luciferase named NanoLuc (NLuc) was engineered and developed from the luciferase gene of deep-sea shrimp *Oplophorus Gracilirostris* [35,36]. Using this luciferase, NanoLuc Binary Technology (NanoBiT) was developed, which is a two-subunit system that can be used to detect PPIs. In this system, Large BiT (LgBiT; 17.6 kDa) is complemented by small BiT (SmBiT; 1.3 kDa), and emits bioluminescence over 150 times stronger than conventional firefly luciferases [37]. To construct biosensors for detecting protein–protein interaction, LgBiT and SmBiT subunits are fused to proteins of interest. When these fusion proteins interact, the NanoBit subunits come into close proximity, causing LgBiT and SmBiT complementation, and the generation of a bright, luminescent signal in the presence its substrate furimazine (Figure 1A).

In this study, we used NanoBiT technology to create a new, highly sensitive, stable, and ultra-bright NanoLuc-YAP/TAZ-TEAD bioluminescent biosensor (BS) by fusing SmBiT and LgBiT to the N-terminus of YAP/TAZ and TEAD1, respectively. We further confirmed the specificity of the biosensors by mutations, and we successfully validated their activity in living cells and in vitro using purified proteins. We then subjected the YAP–TEAD biosensor to an HTS of a library of 2688 small molecules and identified a compound which disrupts YAP–TEAD interaction. Our data convincingly demonstrate that this novel biosensor can be used as a sensitive, simple, fast, cheap, and potent tool for high throughput small molecule screening using purified proteins. It may also hold value for basic science research aimed at delineation of the Hippo signaling network.

## 2. Results

### 2.1. Design and Development of a Highly Sensitive YAP/TAZ–TEAD Biosensor

As previously described, the YAP transcriptional co-activator interacts with the TEAD family of transcription factors (i.e., TEAD1–4) in the nucleus to regulate cell proliferation and survival through transactivation of downstream genes. The crystal structures of YAP and TEAD1 have been resolved which indicate that YAP residues 50–171 complex with TEAD residues 194–411 [38]. Therefore, we constructed a biosensor based on these interacting YAP and TEAD fragments. In order to determine the optimal orientation for a YAP-TEAD biosensor, eight constructs were made with LgBiT and SmBiT domains positioned at the N- and C-termini of YAP or TEAD fragments (Figure S1). These constructs were co-expressed in HEK293T cells, lysed with passive lysis buffer and NanoBiT assays were performed. As presented in Figure 1B, all combinations of SmBiT and LgBiT biosensors showed relatively high luminescent signal and activity, but the combination of SmBiT–YAP and LgBiT–TEAD1 showed the highest sensitivity, dynamic range, and activity. Therefore, it was selected for further experiments. Using a similar strategy, and with the same orientation as YAP, we designed a biosensor to detect the interaction of residues 13-119 of TAZ (a YAP paralog) with TEAD. Of note, we did construct biosensors with full-length YAP and TEAD cDNAs however these biosensors generated bioluminescent signals that were too low to be of value in further experiments.

### 2.2. Validation of YAP/TAZ–TEAD–BS In Vivo and In Vitro

**Figure 2:**
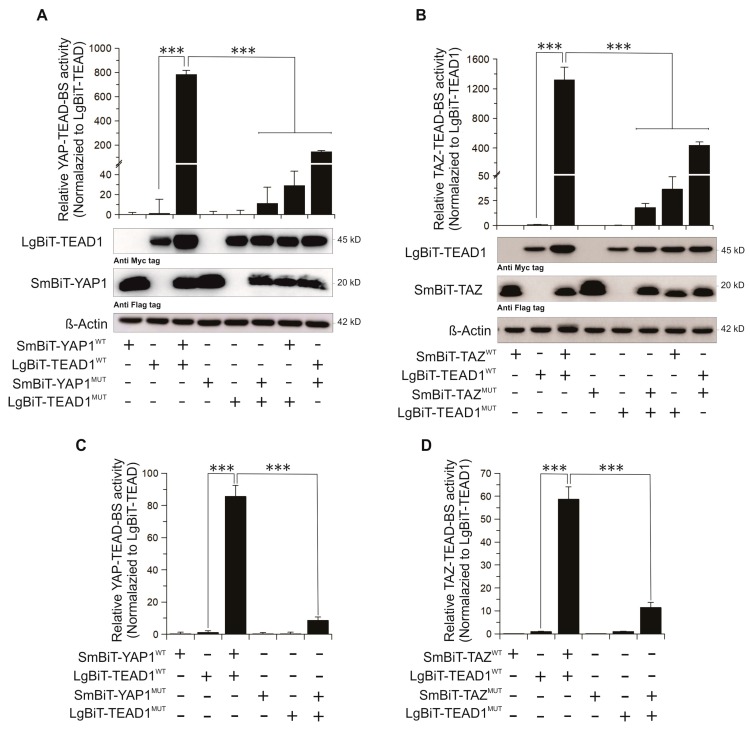
Validation of YAP–TEAD and TAZ–TEAD biosensors in the cells (**A**,**B**) and as purified fusion protein in vitro (**C**,**D**). Specificity of the biosensors was tested by applying multiple point mutations in each pair of biosensors. (**A**,**B**) Each pair of biosensors was transfected into HEK293T cells. Biosensor activity and expression of LgBiT-TEAD (using anti-Myc tag antibody), SmBiT-YAP and SmBiT-TAZ (using anti-Flag tag antibody) was determined 48 h after transfection. (**C**,**D**) Biosensors were purified as His-tagged fusion proteins from *Escherichia coli* and 100 ng of each construct was used to test the activity of YAP–TEAD (**C**) and TAZ–TEAD (**D**) biosensor. Proteins for each pair of biosensors were mixed and incubated for 5 min, the substrate was added and luminescence was measured. Mean ± SD; *n* = 3; ***, *p* < 0.001.

We next validated our YAP/TAZ–TEAD biosensors both in living cells and in vitro using purified proteins. HEK293T cells were transfected with equal amounts of each biosensor component plasmid. After 48 h, the cells were lysed and NanoBiT assays were performed in which the activity of the YAP/TAZ–TEAD biosensors compared to the basal activity of the LgBiT–TEAD construct alone (Figure 2A,B). To determine the specificity of the biosensors, critical residues in the YAP/TAZ-TEAD interaction domain including M86, R89, L91, S94, F95, F96 in YAP; W43, K46, L48, S51, F52/53 in TAZ; and E255, V257, I262 in TEAD1 [38] were mutated to alanine. Combinations of wild type (WT) or mutant (MUT) SmBiT-YAP/TAZ (SmBiT-YAP/TAZ^WT^ or SmBiT-YAP/TAZ^MUT^) constructs were co-transfected with WT or MUT LgBiT–TEAD1 (LgBiT–TEAD1^WT^ or LgBiT–TEAD1^MUT^), and biosensor activity was assessed using NanoBiT assay and western blotting (Figure 2A,B). As shown in this figure, individual mutations to one biosensor component significantly diminished biosensor activity. Mutation of both biosensor components in the constructs nearly abolished biosensor activity.

We next purified His-tagged wild type and mutant SmBiT–YAP/TAZ and LgBiT–TEAD fusion proteins (40–50% purity) and evaluated their activity in vitro. Consistent with the in vivo experiments, the SmBiT–YAP^WT^ or TAZ^WT^ and LgBiT–TEAD^WT^ showed high luminescent signal in vitro as purified fusion proteins whereas mutation of either component protein significantly reduced biosensor activity. Mutation of both biosensor components diminished luciferase activity to levels comparable with background signal (Figure 2C,D). Thus, we have developed specific YAP–TEAD and TAZ–TEAD biosensors that can be utilized in vivo or in vitro.

**Figure 3:**
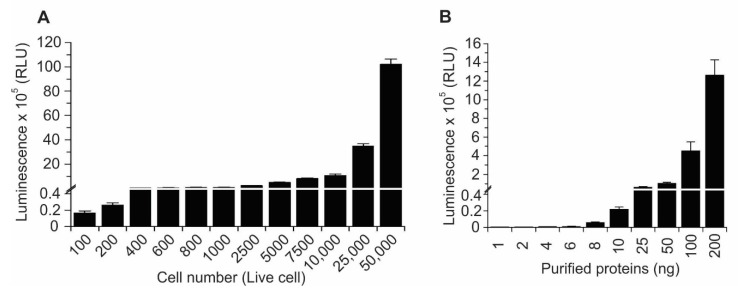
Sensitivity of the YAP–TEAD biosensor. (**A**) After transfecting HEK293T with biosensor, different numbers of cells (100–50,000) were cultured in 96-well plates. After adding NanoBiT substrate, luminescence was measured. (**B**) Different concentrations (1–200 ng) of fusion proteins of each pair of biosensors were combined and biosensor activity was measured. Data are represented as mean ± SD (*n* = 8).

As YAP and TAZ are paralogs with similar structures and functions, we chose to limit our further investigation to the YAP–TEAD biosensor. We set out to determine whether this biosensor would be suitable for use in the identification of compounds inhibiting the YAP–TEAD interaction. We found that this biosensor was extremely sensitive, requiring only 100 live cells to generate a measurable bioluminescent signal in a 96-well plate (Figure 3A). We further measured the sensitivity of our biosensor by using purified fusion proteins. As shown in Figure 3B, bioluminescence was detectable even when only 8 ng of each biosensor component was combined. These results convincingly demonstrate that our YAP–TEAD biosensor is very sensitive and may require only small amounts of cells or purified protein for successful implementation in HTS.

### 2.3. Identification and Validation of Novel Small Molecule Drugs Disrupting the YAP–TEAD Interaction Using the YAP–TEAD Biosensor

**Figure 4:**
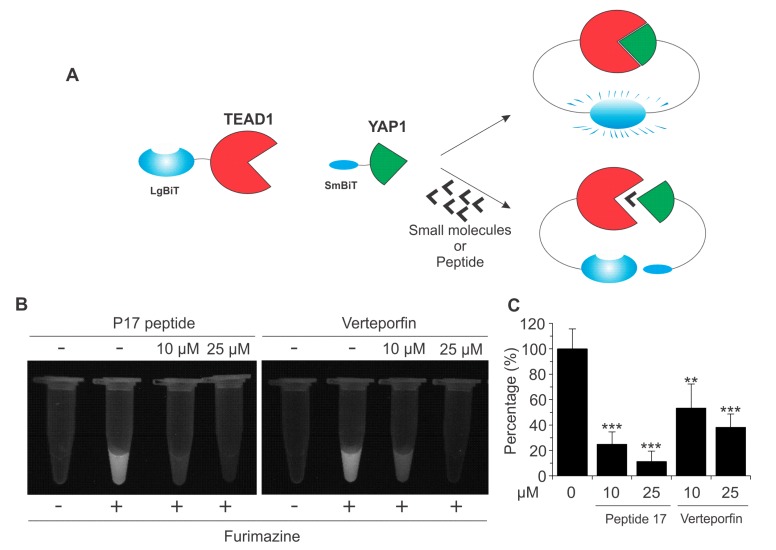
The YAP–TEAD biosensor as a tool for small molecule screening. (**A**) Schematic illustration of disruption of SmBiT–YAP and LgBiT–TEAD by peptide 17 (P17) and Verteporfin. (**B**) Validation of biosensor using P17, and Verteporfin using fusion proteins. In this experiment, the two biosensor components (100 nM each) were combined in the presence of Furimazine and/or peptide 17 or Verteporfin and bioluminescent imaging (BLI) was performed. (**C**) Validation of biosensor using P17, and Verteporfin using fusion proteins. LgBiT–TEAD fusion protein (100 ng) was incubated with P17 or Verteporfin at 4 °C overnight, prior to the addition of Sm–BiT–YAP and NanoBiT luciferase assay. Data are represented as mean ± SD (*n* = 5). **, *p* < 0.01; ***, *p* < 0.001.

We next performed a small-scale screen testing the effects of small molecule inhibitors on the purified YAP–TEAD biosensor fusion proteins in vitro. When compared to in vivo screens in cells, in vitro screening methods using purified proteins generally have less complexity and identify direct inhibitors of PPIs rather than compounds that indirectly affect this interaction through targeting upstream regulators [20,39]. Therefore, despite the success of our YAP–TEAD biosensor in living cells and in vitro using fusion protein, we decided to proceed with in vitro screening to circumvent non-specific interactions and indirect influences of the drugs. As positive controls, we first validated the YAP–TEAD biosensor using the aforementioned known YAP–TEAD inhibitors P17 and VP using bioluminescent imaging (BLI)—a method of non-invasive, direct visualization of bioluminescent signal that can be performed in live cells—and in vitro luciferase assays (Figure 4A–C). As depicted in Figure 4, in addition to the significant reduction of YAP–TEAD biosensor activity measured by NanoLuc luciferase assays, the effects of these inhibitors were visualized very effectively by BLI.

**Figure 5:**
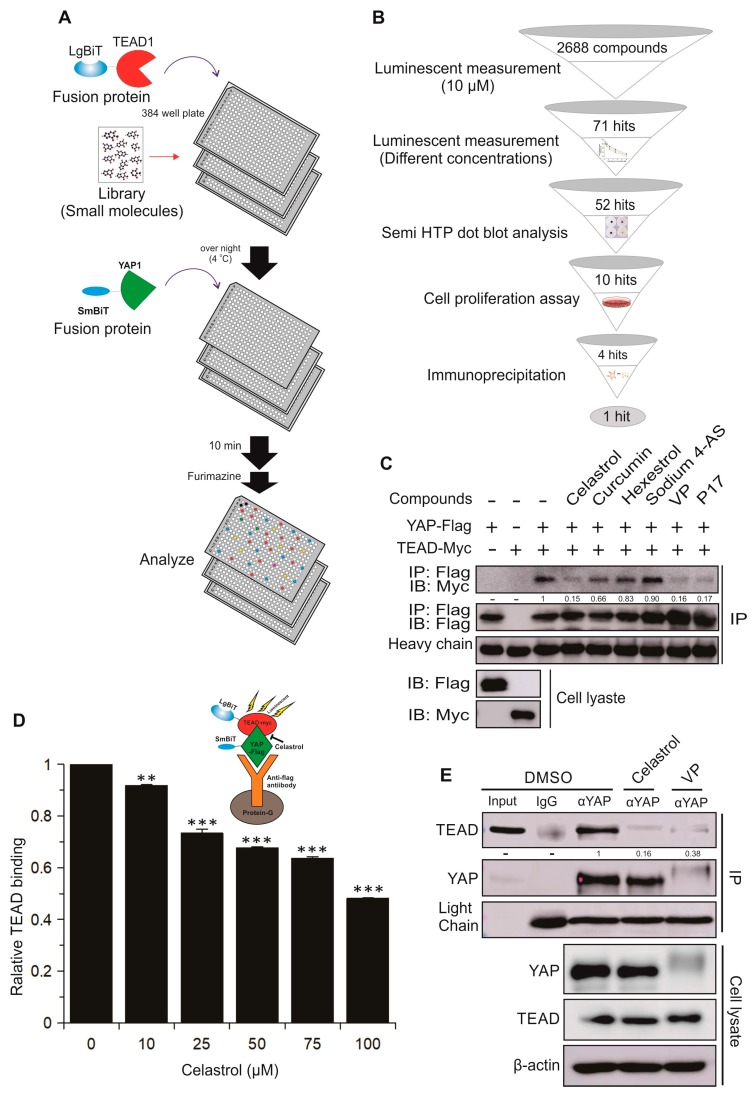
Use of the YAP–TEAD biosensor in drug discovery screening. (**A**) Experimental design for HTS using YAP–TEAD biosensor fusion proteins. LgBiT–TEAD1 fusion protein was distributed into 384-well plates with 10 µM spectrum library and incubated for overnight at 4 °C. The following day, SmBiT–YAP was added and allowed to incubate before addition of NanoBiT substrate (Furimazine) and luciferase assay. (**B**) Protocol for sequential validation of candidate YAP–TEAD inhibitors identified in HTS. (**C**) HEK293T cells were transfected with LgBiT-TEAD1-Myc or SmBiT-YAP1-Flag plasmids and YAP-TEAD binding was assessed by co-immunoprecipitation. LgBiT-TEAD1-Myc lysates were pre-incubated with 100 μM of four different compounds for 1 h before adding SmBiT-YAP-Flag lysate. LgBiT-TEAD1: SmBiT-YAP1 complexes were co-immunoprecipitated with anti-Flag antibody. Co-immunoprecipitated TEAD was quantified by western blotting (Myc antibody). Verteporfin and P17 were used as positive controls. (**D**) Schematic illustration shows the YAP–TEAD NanoBiT interaction/co-immunoprecipitation assay in the presence of Celastrol. Celastrol inhibits SmBiT–YAP/LgBiT–TEAD interaction in a dose-dependent manner. Cell lysates were incubated with different concentrations of Celastrol and co-immunoprecipitation was performed as described in (**C**). NanoBiT assay was performed to assess the relative binding of TEAD to YAP. The data is mean ± SD (*n* = 2). Statistical significance compared to control: **, *p* < 0.01; ***, *p* < 0.001. (**E**) Assessing interaction of endogenous YAP with TEAD using co-immunoprecipitation. H1299 cells were treated with 25 µM Celastrol or Verteporfin as positive control for 4 h. YAP–TEAD complexes were immunoprecipitated using anti YAP antibody with IgG as negative control. Anti-TEAD antibody was used to quantify co-immunoprecipitated TEAD by western blotting. Numbers in western blots represent normalized TEAD/YAP signal density.

We then proceeded with a high throughput small molecule drug screen using a library of 2688 small molecules to identify novel compounds that target the YAP–TEAD interaction. The biosensor assays were adapted to a 384-well-based format (Figure 5A). Z-scores for these assays were generated which exceeded 0.8 and met the universally accepted HTS criteria, indicating robust assay performance under screening conditions. As schematically shown in Figure 5B and Figure S2A, the 71 compounds that reduced luciferase bioluminescence of the YAP–TEAD biosensor at least two-fold were considered primary hits. These initial candidate YAP–TEAD inhibitors from our screen were then subjected to secondary screens using increasing concentrations (0.625–10 μM) of each compound. This screening refined the list of candidate inhibitors to 52 compounds. Thirty-three of these 52 compounds were commercially available, which were purchased from Selleck Chemical (Houston, TX, USA) for additional investigation. We further refined this list to 10 compounds by semi-HTP dot blot analysis (Figure S2B). In this method, GST-YAP (full length) was dotted onto nitrocellulose membranes and incubated in the presence of inhibitor and cell lysate containing full-length TEAD1-Flag, allowing the YAP–TEAD interaction to be visualized by western blotting with anti-Flag antibodies. This validation step was critical in excluding false positive compounds that may had non-specific quenching effects on luciferase rather than the YAP–TEAD interaction.

The effects of the remaining 10 compounds were tested in cell proliferation assays using different cancer cell lines (Figure S2C,D). In these experiments, four compounds were found to decrease cell proliferation/viability (Celastrol, Curcumin, Hexestrol, and Sodium 4-aminosalicylate (Sodium 4-AS)). To test whether these compounds indeed can inhibit YAP-TEAD binding, we performed co-immunoprecipitation assays using cell lysates prepared from HEK293T expressing the biosensors. As shown in Figure 5C, Celastrol, a pentacyclic terpenoid, significantly inhibited binding of SmBiT–YAP to LgBiT–TEAD. Furthermore, we established a YAP–TEAD immunoprecipitation/Nanoluc-based interaction assay to determine the dose-dependent interruption of the YAP-TEAD complex by Celastrol. After transfecting HEK293T cells with either SmBiT-YAP-Flag or LgBiT-TEAD-myc, SmBiT-YAP-Flag proteins were immobilized on Protein-G beads using an anti-Flag antibody and the interaction of SmBiT-YAP-Flag and LgBiT-TEAD-myc was measured using NanoBiT assay in the presence of increasing concentrations of Celastrol. Incubation with Celastrol resulted in a dose-dependent decrease of TEAD bound to YAP and, therefore, decreased luminescent signal, demonstrating that Celastrol inhibited YAP–TEAD1 interaction in a dose-dependent manner (Figure 5D). Since our biosensor constructs are comprised of truncated YAP and TEAD, in order to explore whether Celastrol is able to disrupt a direct interaction of full-length YAP and TEAD, we next performed co-immunoprecipitation experiments with YAP and TEAD expressed at endogenous level in H1299 lung cancer cells. Significantly, similar to the known YAP–TEAD inhibitor VP, we showed that Celastrol is indeed able to interrupt the strong interaction between endogenous YAP and TEAD (Figure 5E).

**Figure 6:**
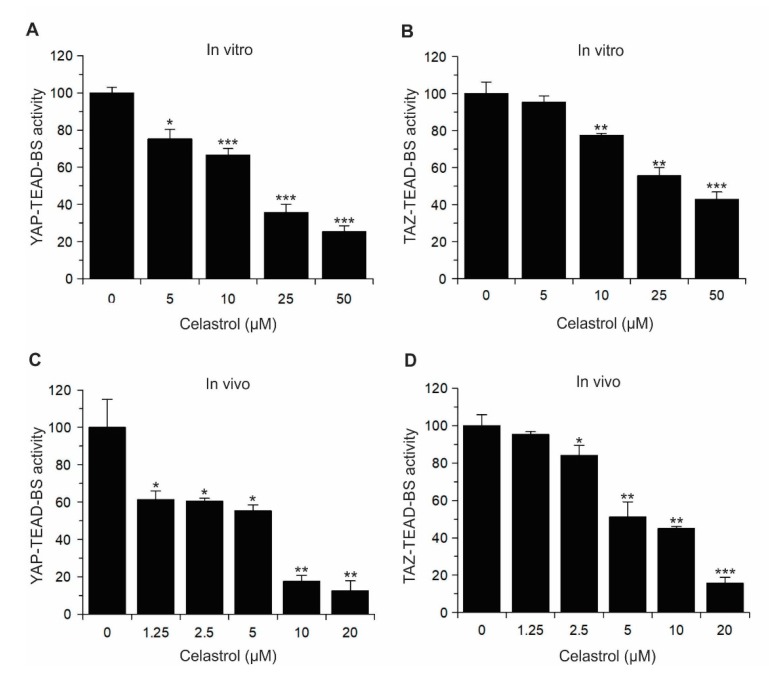
Celastrol inhibits YAP/TAZ–TEAD biosensor activities in vitro and in vivo. Dose-dependent effect of Celastrol on YAP–TEAD (**A**) and TAZ–TEAD (**B**) biosensor activities in vitro. Here, 100 ng of Lg-TEAD fusion protein was incubated with different concentrations of Celastrol overnight at 4 °C, 100 ng of Sm-YAP or Sm-TAZ was added into each well and incubate at room temperature for 5 min. After adding 10 µL NanoGlo Live Substrate (1:25 dilution), luciferase activity was measured using GloMax Navigator Microplate Luminometer. (**C**,**D**) Dose-dependent effect of Celastrol on YAP–TEAD (**C**) and TAZ–TEAD (**D**) biosensor activities in cells. HEK293T cells were transfected with each pair of biosensors, and 48 h later cells were treated with increasing concentration of Celastrol. After 5 h, 10 µL NanoGlo Live Cell Reagent (1:25 diluted) containing furimazine substrate was added and luciferase activity was measured. Data represents mean ± SD (*n* = 3). Statistical significance compared to non-treated cells: *, *p* < 0.05; **, *p* < 0.01; ***, *p* < 0.001.

Furthermore, we tested the effect of Celastrol on not only YAP–TEAD but also TAZ–TEAD biosensor activity both in vitro using fusion proteins (Figure 6A,B) and in live cells (in vivo) (Figure 6C,D). Interestingly, both in vitro and in vivo results showed that Celastrol inhibits both YAP-TEAD-BS and TAZ-TEAD-BS activities. These results demonstrate that the YAP–TEAD and TAZ–TEAD biosensors can be used to identify compounds specifically targeting these interactions in HTS and that Celastrol is a novel inhibitor of the YAP/TAZ–TEAD interaction.

### 2.4. Celastrol Inhibits TEAD-Dependent Target Genes Expression, Cell Proliferation, Cell Viability, Migration, and Colony Formation in Lung and Breast Cancer Cell Lines

**Figure 7:**
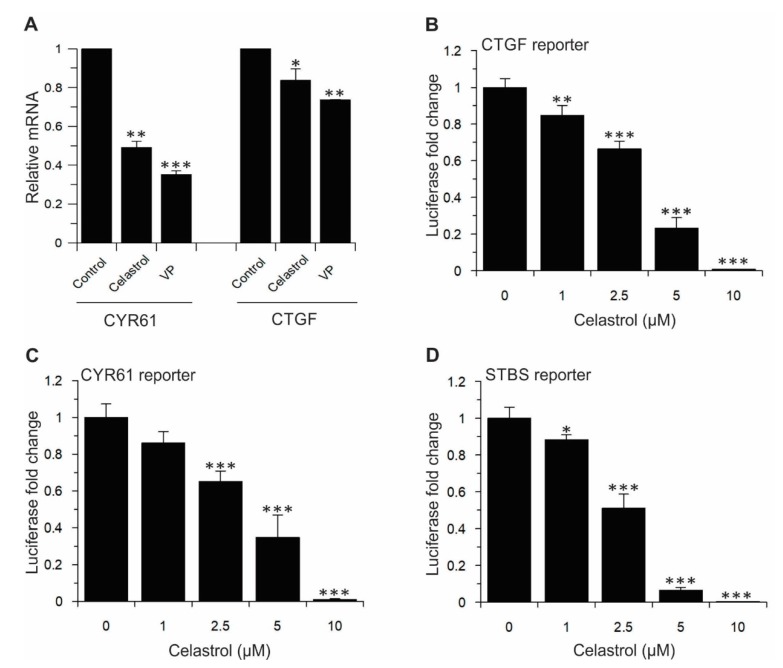
Celastrol significantly affects the expression of Hippo pathway downstream transcriptional target genes. (**A**) Celastrol decreases YAP/TAZ transcriptional coactivation of CTGF and CYR61 in H1299 cells. Cells were treated with 1 µM Celastrol for 12 h. CTGF and CYR61 expression was measured by qRT-PCR (*n* = 2). Data are represented as mean ± SD. (**B**–**D**) The activity of CTGF-Luc, CYR61-Luc, and STBS-Luc reporters were measured in the presence of different concentrations of Celastrol. Cells were transfected with reporters. After 24 h, transfected cells were treated with indicated concentrations of Celastrol. After 12 h of treatment, cells were lysed and luciferase assays were performed. Data are represented as mean ± SD (*n* = 4). *, *p* ≤ 0.05; **, *p* < 0.01; ***, *p* < 0.001.

As previously described, activation of TEAD transcription factors in response to YAP/TAZ binding prompts the expression of numerous genes that are important for cell proliferation, migration and tissue growth. The best characterized TEAD target genes associated with the promotion of cell proliferation, cell motility and migration are *CTGF* and *CYR61* [11,40]. Therefore, we assessed the effect of Celastrol on these genes. Our results showed that the expression of steady-state mRNA levels for both CTGF and CYR61 was significantly inhibited by Celastrol (Figure 7A). In addition, TEAD-dependent reporter gene assays were performed which showed that Celastrol dose-dependently inhibited CTGF, CYR61, and STBS luciferase reporter gene activities (Figure 7B–D).

**Figure 8:**
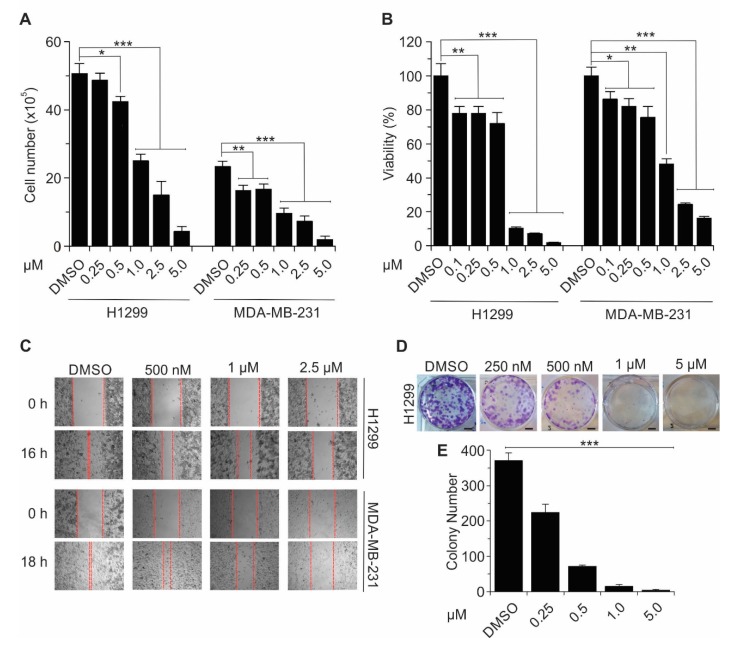
Celastrol inhibits cell proliferation, cell viability, cell migration, and colony formation in lung and breast cancer cells. (**A**,**B**) Celastrol decreases cell proliferation (**A**) and cell viability (**B**) in H1299 and MDA-MB-231 cell lines in a dose dependent manner (concentrations as indicated; total treatment time 72 h). (**C**) H1299 and MDA-MB-231 cells were treated with increasing concentrations of Celastrol and cell migration (wound healing) assay was performed. Representative images (taken at 40x magnification) are shown. (**D**,**E**) Colony formation of Celastrol (0.25. 0.5, 1, and 5 μM; 10 days) treated H1299 cells as compared with nontreated control. Scale bar: 5 mm. Plotted values represent mean ± SD (*n* = 3), *, *p* ≤ 0.05; **, *p* < 0.01; ***, *p* < 0.001.

We next explored the functional significance of inhibition of YAP–TEAD interaction by Celastrol. Celastrol significantly inhibited cell proliferation (Figure 8A) and decreased cell viability (Figure 8B) in H1299 lung and MDA-MB-231 breast cancer cells. Similarly, cell migration was dramatically inhibited in the presence of Celastrol (Figure 8C). Finally, Celastrol significantly inhibited cell growth in H1299 (up to 88% reduction at 5 μM) (Figure 8D,E). These results confirmed that Celastrol is a potent inhibitor of various cellular processes that are mediated by YAP/TAZ–TEAD binding.

## 3. Discussion

In recent years, a sparsity of available tools has impeded the measurement of the dynamics and activity of the Hippo pathway core components in a quantitative, high-throughput and non-invasive manner. In the present study, we have developed the first ultra-bright NanoBiT bioluminescent biosensors for quantifying a critical interaction for Hippo pathway-related tumorigenesis: YAP/TAZ–TEAD interaction. Since the Hippo pathway plays critical roles in various biological processes, these biosensors can be easily utilized in monitoring the dynamics of YAP/TAZ–TEAD interaction in both drug discovery and basic science research.

Historically, a variety of methods and strategies, such as fluorescence resonance energy transfer (FRET), Thermal Shift Assay, AlphaLisa assay, SPR, and in Silico Molecular Docking have been used to study PPIs. Compared to these methods, our newly developed biosensors monitoring YAP/TAZ–TEAD PPIs have several key advantages. First, they are ultrasensitive with signals detectable in as few as 100 cells. Second, the YAP–TEAD PPI can be visualized using BLI to reveal the effect of drugs on YAP–TEAD activity. Additionally, these biosensors are extremely stable. They can be purified and used for quantification of YAP/TAZ–TEAD PPIs in vitro using only 8 ng of biosensor proteins. Significantly, due to their high sensitivity, our biosensors can be used in vitro in 384-well format to perform large scale HTS of novel small molecule drugs disrupting YAP/TAZ–TEAD PPIs. Indeed, using our biosensor, we performed the first high throughput biochemical screening for small molecules disrupting YAP–TEAD interaction using a library of 2688 small molecule compounds. After extensive validation using various approaches, we identified Celastrol as a small molecule compound directly disrupting the YAP–TEAD PPI in vitro and in vivo. Although Celastrol has been used as a drug inhibiting cancer growth through several signaling pathways [41], this is the first demonstration that it can directly interact with and disrupt YAP/TAZ–TEAD interaction both in vitro and in vivo. In our vast experimentation using these biosensors, we have found them to be very stable and functional even at room temperature although we performed our screening at 4 °C. Moreover, this system could be scaled-up to accommodate larger-scale screens to identify more candidate small molecule drugs targeting YAP/TAZ–TEAD. Indeed, it is conceivable that this biosensor could screen thousands of compounds per day in a 384-well plate arrangement, requiring relatively small amounts of protein for each reaction.

## 4. Materials and Methods

### 4.1. Plasmid Construction

To make fusions of YAP (aa 50–171), TAZ (aa 13–119), TEAD1 (aa 194–411), and their mutants with LgBiT and SmBiT in pcDNA3.1/hygro vector, tandem, and overlapping PCR were performed using different TK vectors (Promega) and full-length cDNA of YAP (accession number NM_006106.4), TAZ (accession number NM_001168278.2), and TEAD1 (accession number NM_021961.6) as templates. The amplified PCR products were cloned into the BamHI/NotI sites of pcDNA3.1/hygro(+). To make His-tagged fusion proteins, cDNAs of SmBiT-YAP1, SmBiT-TAZ, LgBiT-TEAD1, and their mutants were amplified by PCR using respective constructs cloned in pcDNA3.1 vector as template, and subsequently cloned into the BamHI/NotI sites of pET23b. To make GST-YAP, DNA coding full length YAP was cloned into the BamHI/NotI sites of pGEX-4T1. See supporting information for primers used in cloning (Table S1).

### 4.2. Cell Culture

HEK293T (human embryonic kidney) and A549 (human lung adenocarcinoma) cells were cultured in Dulbecco’s modified Eagle’s medium (DMEM; Sigma D6429, Oakville, Canada) containing 10% FBE, and 1% P/S (Invitrogen, CA, USA. H1299 (human non-small cell lung carcinoma) cells were cultured in RPMI-1640 Medium (Sigma #8758) containing 10% FBE and 1% P/S (Invitrogen), 10 mM HEPES, 1 mM sodium pyruvate, and 2.5 mg/Ml glucose. For MDA-MB231 cells, DMEM was supplemented with 10% FBS, 1% P/S, and 1% non-essential amino acids (Sigma M7145). All cells were grown at 37 °C with 5% CO_2_.

### 4.3. NanoLuc Luciferase (NanoBiT) Assay

Here, 3 × 10^5^ HEK293T cells were plated in triplicate 24 h before transfection. SmBiT-YAP/TAZ and LgBiT-TEAD1 plasmids were transfected alone (250 ng each) or together into HEK293T cells using PolyJet transfection reagent (SignaGen, Rockville, MD, USA). After 48 h, cells were lysed using passive lysis buffer (Promega, WI, USA) and NanoLuc luciferase assay was performed using Nano-Glo Live Cell Reagent (Promega). For live cell analysis, biosensors were transfected into HEK293T cells and after 24 h, cells were trypsinized, and different numbers of the cells (100–50,000) were seeded into a 96-well plate. After 24 h, Nano-Glo Live Cell Reagent, containing the cell-permeable furimazine (substrate), was added to each well and luminescence was measured. Results are presented as RLU (Relative Luminescence Unit) or fold change compared to control. To study the dose-dependent effect of Celastrol on YAP/TAZ–TEAD biosensor activity in cells, 5 h after transfection in six-well plates, cells were trypsinized and 2 × 10^4^ cells/100 μL were seeded into each well of a 96 well plate. Then, 40 h later, the medium was replaced with increasing concentration of Celastrol (0, 0.125, 2.5, 5, 10 μM in 100 μL media). After 5 h, the medium was aspirated and 50 μL 37 °C warmed Opti-MEM medium was added to each well. Then, 10 μL Nano-Glo Live Cell Reagent (1:25 diluted) containing cell-permeable furimazine substrate was added, and the biosensor activity was immediately measured using GloMax Navigator Microplate Luminometer (Promega, WI, USA).

In the case of fusion proteins, a defined amount of each pair of proteins was mixed and, after adding the substrate, NanoLuc luciferase assay was performed using Turner Biosystems 20/20 luminometer (Promega, WI, USA) or GloMax Navigator Microplate Luminometer (Promega, WI, USA). To evaluate the dose-dependent effects of Celastrol on the TAZ–TEAD biosensor activity in vitro, 100 ng of LgBiT-TEAD-His in 40 µL/well buffer [30 mM Tris-HCl, pH 7.5, 150 mM NaCl, 5 mM MgCl_2_, and 3 mM DTT)] in a 96-well plate was incubated with increasing concentrations of Celastrol for overnight at 4 °C. The next day, 100 ng of SmBiT–YAP or SmBiT–TAZ fusion protein was added into the 96-well plate and incubated for 5 min. Then, 10 µL NanoGlo Live Substrate (1:25 dilution from stock) was added into each well, mixed, and luciferase activity was immediately measured using the GloMax Navigator Microplate Luminometer.

### 4.4. Cell Proliferation Assay

To evaluate the effects of 10 “hit” compounds on cell proliferation, 2 × 10^4^ cells (H1299 and A549) were seeded in triplicate in 24-well plates and after 24 h were treated with 1 µM of each small molecule. After four days, cells were counted. To investigate dose dependent effect of Celastrol on cell proliferation, 4 × 10^4^ H1299 and MDA-MB-231 cells were seeded in triplicate into each well of 12-well plates. After 24 h, cells were treated with different concentrations (0.25, 0.5, 1, 2.5, and 5 µM) of Celastrol and counted three days after treatment. Data are shown as mean ± S.D.

### 4.5. Cell Viability Assay

Cell viability determination was performed using CellTiter-Glo^®^ 2.0 Assay kit (Cat #G9242) according to the manufacturer’s protocol from Promega.

### 4.6. Clonogenic Assay

Here, 5 × 10^2^ H1299 cells were seeded in six-well plate. The next day, cells were treated with different concentrations of Celastrol. After 10 days, cells were washed with cold PBS, fixed with pure cold methanol for 2 min, and then stained for 5 min with staining solution containing 0.05% crystal violet in 25% methanol.

### 4.7. Wound Healing Assay

Here, 2 × 10^5^ H1299 and MDA-MB-231 cells were seeded in 24-well plates (Ibidi #82406, Gräfelfing, Germany) and grown until 100% confluent. A wound was generated by removing the insert according to company protocol and cells were treated with 0.5, 1, and 2.5 µM Celastrol. Images were captured using a Nikon TE-2000U (Melville, NY, USA) inverted microscope after 16 and 18 h of treatment for H1299 and MDA-MB-231 cells, respectively.

### 4.8. Western Blotting and Antibodies

Extracted samples were separated by 12% SDS-polyacrylamide gel electrophoresis (PAGE) (Bio-Rad) and transferred to nitrocellulose membranes. Antibodies for western blot analysis were as follows: mouse monoclonal anti-Myc (9E10) from Roche (Basel, Switzerland); mouse monoclonal anti-Flag (F1804) and mouse monoclonal anti-β-actin (AC-15) from Sigma; mouse monoclonal YAP antibody (63.7) from Santa Cruz Biotechnology (Dallas, TX, USA) and mouse monoclonal TEAD4 (ab58310) from Abcam (Cambridge, United Kingdom). First antibody incubation was followed by probing with the corresponding secondary antibody and developing using Amersham Imager (Little Chalfont, United Kingdom). Uncropped images of western blottings are available in Figure S3.

### 4.9. Semi HTP Dot Blot Analysis of Inhibition of Small Molecule Drugs on YAP–TEAD Interactions

For semi HTP dot blot analysis, HEK293T cells were transiently transfected with TEAD1-Myc. After 48 h, cells were harvested and lysed in 1% NP40 lysis buffer. GST-YAP fusion protein (500 ng in 2 µL) was placed on nitrocellulose membrane as a dot and allowed to dry for 5 min at room temperature. The membrane was then incubated in buffer (30 mM Tris-HCl, pH 7.5, 150 mM NaCl, 5 mM MgCl_2_, and 3 mM DTT) overnight at 4 °C with or without small molecule compound (10 µM). The next day, the buffer was aspirated and cell lysate containing TEAD1-Myc (20 µg in 200 µL), pre-incubated with small molecules, was added. After 2 h incubation, the membrane was washed three times (5 min) with 1% NP40 lysis buffer followed by western blotting using anti-myc antibody.

### 4.10. Co-Immunoprecipitation

For co-immunoprecipitation using biosensor, HEK293T cells were transiently transfected with either TEAD1-Myc or YAP-Flag. Forty-eight hours after transfection, cells were harvested and lysed in 1% NP40 lysis buffer, and cell extracts were pre-cleared with protein G beads overnight. Then, 400 µg of TEAD-Myc-containing extracts were incubated with 100 μM of different compounds in 300 µL for 1 h at 4 °C. An equal amount and volume of YAP-Flag-containing lysate was added, and the mixture was incubated for a further 1 h at 4 °C. Two µg of anti-flag antibody was added and incubated for 2 h to immunoprecipitate YAP-Flag, followed by the addition of protein G beads to the tube and incubation for one additional hour. Following washing four times with 1% NP40 lysis buffer, immunoprecipitated YAP–Flag proteins were eluted by boiling in 1× SDS buffer and analyzed by western blotting using anti-Flag antibody.

For the YAP−TEAD interaction assay in the presence of different concentrations of Celastrol, co-immunoprecipitation was performed as described above; however, cell lysates were pre-treated with the indicated concentrations of Celastrol in NP40 lysis buffer. In the last step, after four washes, bound LgBiT-TEAD-Myc was detected and quantified by NanoBiT assay.

In the case of co-immunoprecipitation of endogenous YAP and TEAD, H1299 lung cancer cells were treated with 25 µM Celastrol or Verteporfin for 4 h and were lysed using 1% NP40 lysis buffer. For each sample, 2 mg cell lysates were pre-cleared with protein A beads overnight. Two µg of anti-YAP antibody or IgG as negative control was added to each tube and incubated for 4 h to immunoprecipitate endogenous YAP, followed by adding protein A beads to the tubes and incubation for two more hours. Samples were washed four times with 1% NP40 lysis buffer, boiled in 1× SDS buffer and analyzed by western blotting using anti-TEAD antibody.

### 4.11. Bioluminescence Imaging (BLI)

To visualize YAP–TEAD biosensor activity and determine if the biosensor could be used to measure response to inhibitors, 100 nM of fusion proteins were mixed in the presence or absence of Peptide 17 and Verteporfin as inhibitors. After 20 min, furimazine was added, and BLI was performed. Imaging was performed using a biophotonics system, and images were acquired using Image Pro Plus software as previously described [31].

### 4.12. Fusion Protein Purification

CodonPlusRIPL or BL21DE3 *Escherichia coli* strains were transformed and used to purify the fusion proteins. All proteins were purified as either GST or His-tagged fusion proteins according to the protocols with slight modifications from what was previously described [42]. After inoculation, when the OD_600_ was between 0.6–0.8, protein expression was induced with 0.3 mM IPTG (isopropyl β-D-1-thiogalactopyranoside) overnight at 20 °C. Bacterial cells were lysed by sonication, bacterial lysates were centrifuged to collect soluble fractions, and tagged proteins were isolated from the supernatant via Ni-NTA or GST affinity purification. Proteins were subjected to dialysis over night at 4 °C using a standard buffer (30 mM Tris-HCl, pH 7.5, 150 mM NaCl, 5 mM MgCl_2_, and 3 mM DTT) and, after concentrating, they were analyzed by SDS-PAGE, and stored at −80 °C.

### 4.13. Small Molecule Screen

HTS for 2688 small molecules were performed in the Department of Cancer Genetics and Genomics, Small Molecule core, at the Roswell Park Cancer Institute. Experimental conditions were as follows. 40 µL/well [0.5 µL (100 ng) LgBiT-TEAD-His + 39.5 μL buffer (30 mM Tris-HCl, pH 7.5, 150 mM NaCl, 5 mM MgCl_2_, and 3 mM DTT)) were distributed into 384-well plates. Then, a spectrum library (2688 compounds) was added into a 384 well plate (10 µM) and incubated for overnight at 4 °C. The next day, 2 µL/well (0.5 µL SmBiT-YAP-His (100 ng) + 1.5 µL buffer) was added into the 384-well plate and incubated for 5 min. Biosensor activity was measured after adding 5 µL of NanoGlo substrate proceed to luciferase activity measurement (emission 460 nm). Screening was performed a second time with incubation of drug with SmBiT-YAP-His first, followed by addition of LgBiT-TEAD-His. Compounds that reduced luciferase bioluminescence of the YAP–TEAD biosensor by ≥2 fold were considered primary hits. Secondary screens were performed on these primary hits using the biosensor and five different concentrations (0.625, 1.25, 2.5, 5, 10 µM) of each compound.

### 4.14. Reporter Luciferase Assay

In order to have the same cell source and transfection efficiency for all samples used for each reporter assay, 3 µg of CTGF-Luc, CYR61-Luc, or STBS-Luc reporter were first transfected into HEK293T cells in 100 mm dishes (4 × 10^6^ cells) using Polyjet transfection reagent (SignaGen SL100688) according to the manufacturer’s instructions. The next day, cells were trypsinized, and triplicates of cells were seeded in 24-well plates (2 × 10^5^ cells per well). After 10 h, when cells were completely attached and expanded, the cells were treated with different concentrations of Celastrol (1, 2.5, 5, and 10 µM). Twelve hours after treatment, the cells were lysed using passive lysis buffer (200 µL) and luciferase assays were performed using a Luciferase Assay Kit (Promega) and GloMax Navigator Microplate Luminometer. Luciferase fold-change was calculated based on the ratio of luciferase activity of the reporters in treated cells, to that of non-treated control cells.

### 4.15. RNA Extraction and qRT-PCR

H1299 cells were grown in six-well plates with 50–60% confluency. After 24 h, cells were treated with 1 µM Celastrol or Verteporfin for 12 h. RNA extraction was performed using RNAzol RT reagent (200 μL/well) (Molecular Research Center, Inc., Cincinnati, OH, USA) according to the manufacturer’s instruction. Fifty ng RNA/reaction was used to perform qRTPCR using the SuperScript III Platinum SYBR Green One-Step qRT- PCR Kit (Invitrogen). Then, 18S rRNA was used as an internal control.

### 4.16. Statistical Analysis

Data shown in the graphs are the average of at least triplicate experiments except for qRT-PCR, which was performed in duplicate. Unless otherwise specified, data are expressed as the mean ± S.D. Student’s *t*-test (two-tailed) was used for statistical analysis. A value of *p* ≤ 0.05 was accepted as statistically significant.

## 5. Conclusions

In conclusion, we have successfully developed the first YAP/TAZ–TEAD NanoBiT biosensors that can easily be used to study the YAP–TEAD PPI and screen small molecules that target this complex. Such compounds disrupting YAP–TEAD interaction may be useful for the treatment of Hippo pathway–related diseases (cancer progression, drug resistance, anti-angiogenesis therapy, metastasis, diabetes, and age-related macular degeneration).

## Supplementary Materials

The following are available online at https://www.mdpi.com/2072-6694/11/10/1596/s1, Figure S1. Schematic representation of constructs used for the selection round of the YAP–TEAD biosensor, Figure S2. High Throughput Screening (HTS) using YAP–TEAD biosensor fusion proteins as tool and hits validations, Figure S3. Uncropped images of western blotting, Table S1. List of primers for cloning.
