# Supplementary material for: Identification of Celastrol as a Novel YAP-TEAD Inhibitor for Cancer Therapy by High Throughput Screening with Ultrasensitive YAP/TAZ–TEAD Biosensors
Source: Cancers (Basel). 2019 Oct 19;11(10):1596. doi: 10.3390/cancers11101596 (PMC6826516; doi:10.3390/cancers11101596)
Supplement: Supplementary file 1 [file cancers-11-01596-s001.pdf]

Supplementary Materials: Identification of Celastrol as a Novel YAP-TEAD Inhibitor for Cancer Therapy by High Throughput Screening with Ultrasensitive *YAP/TAZ-TEAD* Biosensors

Kazem Nouri, Taha Azad, Min Ling, Helena J. Janse van Rensburg, Alexander Pipchuk, He Shen, Yawei Hao, Jianmin Zhang and Xiaolong Yang

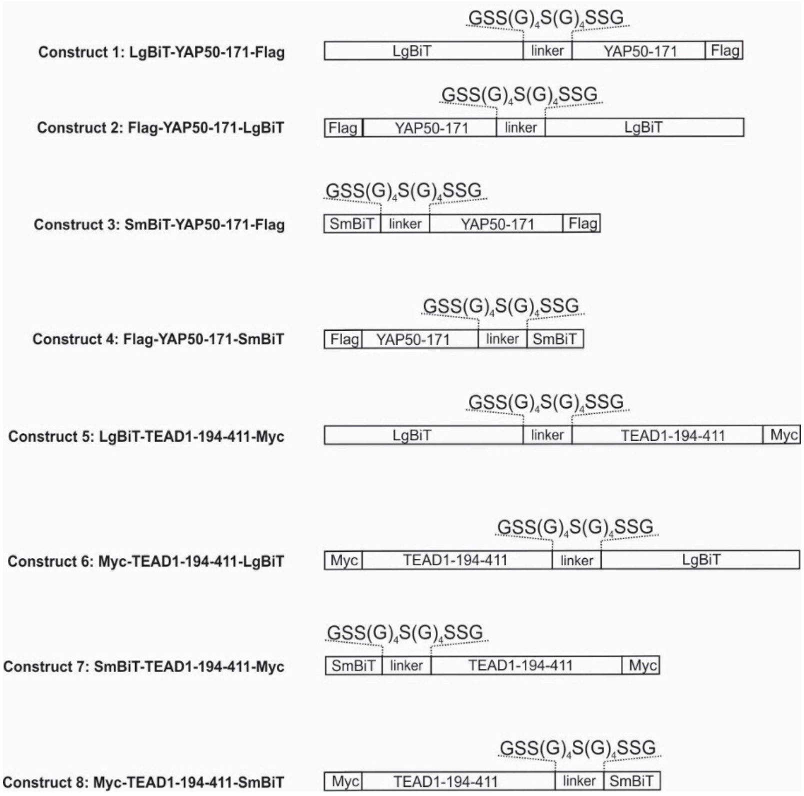

Figure S1. Schematic representation of constructs used for the selection round of the YAP-TEAD biosensor.

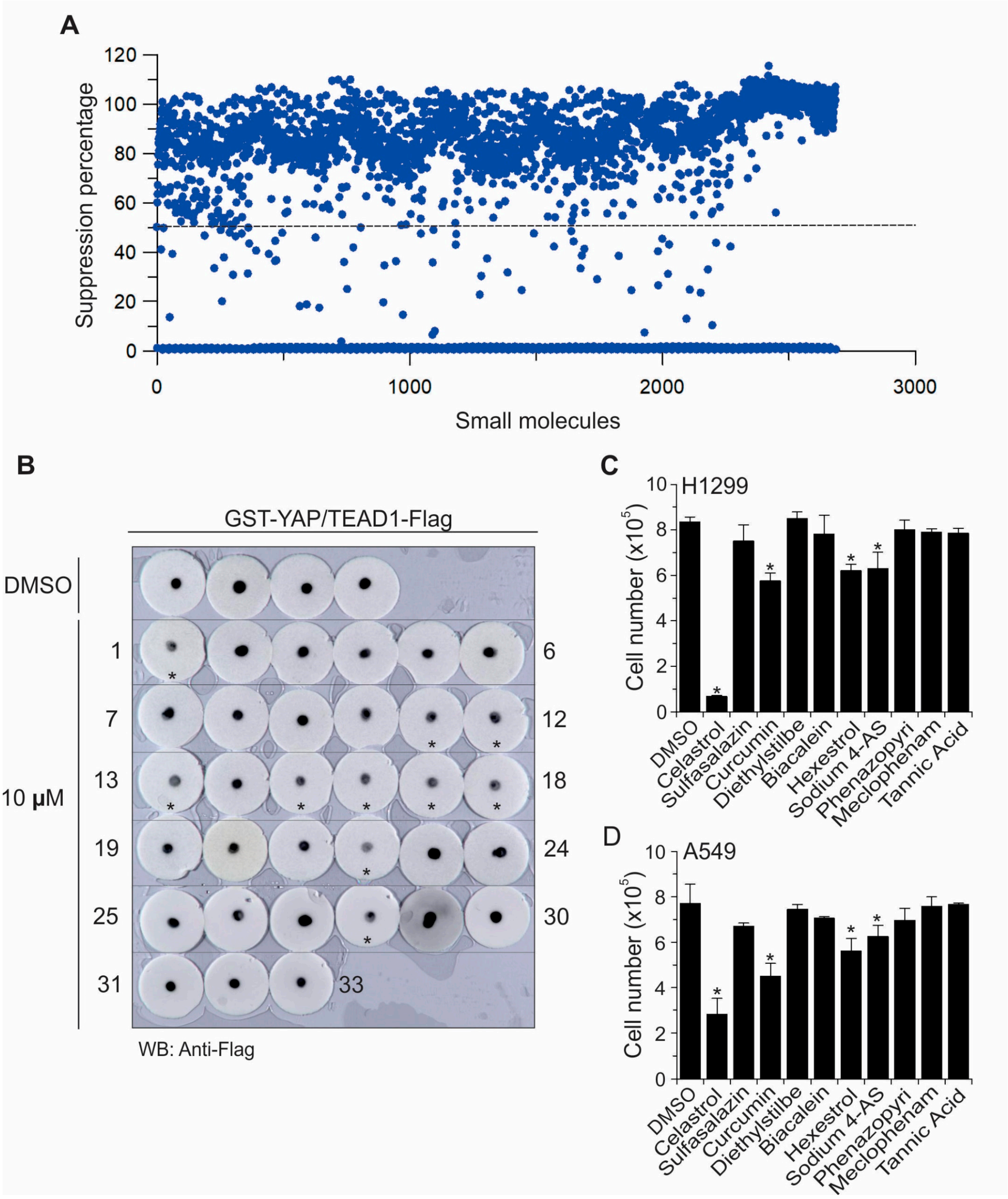

Figure S2. High Throughput Screening (HTS) using YAP-TEAD biosensor fusion proteins as tool and hits validations. (A) HTS using 2688 small molecules in 384-well plates. LgBiT-TEAD1 fusion protein was distributed into the plates, treated with 10 μM spectrum library, and incubated overnight at 4 °C. The next day, SmBiT-YAP was added and proceeded with NanoBiT assay. The dotted line denotes 50% luminescent suppression and those small molecules below the line were considered primary hits and chosen for secondary validation using 5 different concentrations of each compound (data not shown). (B) Semi HTP dot blot analysis of GST-YAP/TEAD1-Flag interaction in the presence of thirty-three different small molecules (10 μM) followed by western blotting using anti-Flag antibody. Asterisk shows small molecules which decrease the interaction of YAP-TEAD and were chosen to proceed with cell proliferation assay as the next validation. (C-D) Cell proliferation assay for H1299 and A549 cells in the presence of 1 μM (4 days) of different small molecules identifies as hits in the semi HTP dot blot analysis. Asterisk shows the small molecules which significantly affect the cell proliferation of both H1299 and A549 cells and these compounds were consider as potential disrupters of the YAP-TEAD interaction and were validated further by co-immunoprecipitation (see Figure 7C). Data are represented as mean ± SD (n = 2). *, statistically significant.

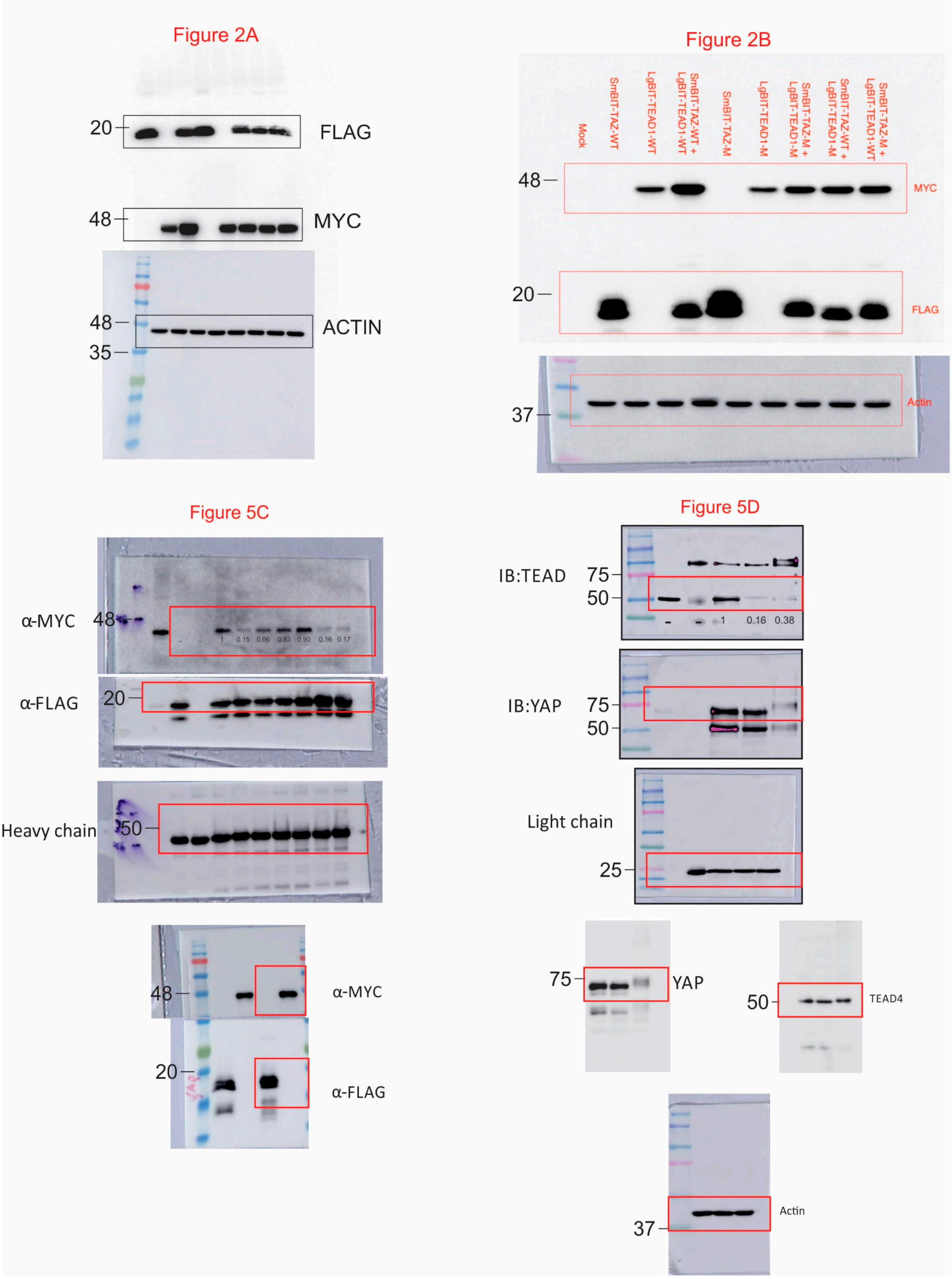

Figure 3. Uncropped western blots from primary figures are shown.

Table S1. List of primers for cloning.

Primer Name	Sequence (5′ to 3′)
B1-Kozak-LgBIT-F	CTGGATCCGCCGCCACCATG GTCTTCACACTCG AAGATTTC
LgBIT -(GS)-R	ACCGCTCGAGCCTCCACCTCCGCTCCCGCCACCACCGGAACTCCCACTGTTGA T
(GS)-YAP50-F	GGGAGTTCCGGTGGTGGCGGGAGCGGAGGTGGAGGCTCGAGCGGTGCCGGG CATCAGATCGTGCACGTC
N1-FLAG-YAP171-R	ATGAAACTGCGGCCGCCTTGTCGTCATCGTCTTTGTAGTCTACATCATCAGGT ATCTCAAAAG
B1-YAP50-F	CTGGATCCGCCGGGCATCAGATCGTGCACGTC
(GS)-YAP171-R	ACCTGACGACCCTCCACCTCCGCTCCCGCCACCACCGCTCGAGCCTACATCAT CAGGTATCTCAAAAG
(GS)-LgBIT-F	GGCTCGAGCGGTGGTGGCGGGAGCGGAGGTGGAGGGTCGTCAGGTGTCTTCA CACTCGAAGA TTTC
N1-LgBIT-R	ATGAAACTGCGGCCGCTTAACTGTTGATGGTTACTCGGAACAG
B1-Kozak -SmiBIT-(GS)-F	CTGGATCCGCCGCCACCATGGTGACCGGCTACCGGCTGTTCGAGGAGATTCTC GGGAGTTCCGGTGGTGGCGGGAGCGGAGGTGGAGG CTCGAGCGGT
(GS)-YAP50-F	GGGAGTTCCGGTGGTGGCGGGAGCGGAGGTGGAGGCTCGAGCGGTGCCGGG CATCAGATCGTGCACGTC
N1-SmBIT-(GS)-R	ATGAAACTGCGGCCGCTTAGAGAATCTCCTCGAACAGCCGGTAGCCGGTCAC ACCTGACGACCCTCCACCTCCGCTCCCGCCACCACCGCTCGAGCC
(GS)-TEAD-194-F	GGGAGTTCCGGTGGTGGCGGGAGCGGAGGTGGAGGCTCGAGCGGTGAGCCT GCATC GGCCCCAGCT CCCTCAG
N1-TEAD411-myc-R	ATGAAACTGCGGCCGCTTACAGATCCTCTTCTGAGATGAGTTTTTGTTCATTTG AAACTTCAAACACACAGGC
B1-TEAD194-F	CTGGATCCGAGCCTGCATC GGCCCCAGCTCCCT CAG
GS-TEAD-411-R	ACCTGACGACCCTCCACCTCCGCTCCCGCCACCACCGCTCGAGCCATTTGAA ACTTCAAACACACAGGC
(GS)-TEAD194-F	GGGAGTTCCGGTGGTGGCGGGAGCGGAGGTGGAGGCTCGAGCGGTGAGCCT GCATC GGCCCCAGCT CCCTCAG
N1-TEAD411-myc-R	ATGAAACTGCGGCCGCTTACAGATCCTCTTCTGA GATGAGTTTTTGTTCATTTGAAACTTCAAACACACAGGC
YAP (50–171)-M86A-R89A-L91A-S94A-F95A-F96A-S	AACGTGCCCCAGACCGTGCCCGCTAGGCTCGCCAAGGCTCCCGACGCCGCTG CAAAGCCGCCGGAGCCCAAATCC
YAP (50–171)-M86A-R89A-L91A-S94A-F95A-F96A-AS	GGATTTGGGCTCCGGCGGCTTTGCAGCGGCGTCGGGAGCCTTGGCGAGCCTA GCGGGCACGGTCTGGGGCACGTT
TEAD1(194–411)-E255A-V257A-I262A-S	TCTTACAGTGACCCATTGCTTGCCTCAGCTGACATTCGTCAGGCTTATGACAA ATTTCCTGAAAAG
TEAD1(194–411)-E255A-V257A-I262A-AS	CTTTTCAGGAAATTTGTCATAAGCCTGACGAATGTCAGCTGAGGCAAGCAAT GGGTCACTGTAAGA
(GS)-TAZ13-F	GGGAGTTCCGGTGGTGGCGGGAGCGGAGGTGGAGGCTCGAGCGGTCCTGGG CAGCAAGTGATCCAC
N1-FLAG-TAZ119-R	ATGAAACTGCGGCCGCCTTGTCGTCATCGTCTTTGTAGTCGTCGTAGGACTGC TGGCGGAG
TAZ-W43A-K46A-L48A-S51A-F52/53A-F	AATCCGAAGCCTAGCTCGGCGCGGAAGGCGATCGCGCCGGAGGCTGCCGCT AAGGAGCCTGAT 3′
TAZ- W43A-K46A-L48A-S51A-F52/53A-R	ATCAGGCTCCTTAGCGGCAGCCTCCGGCGCGATCGCCTTCCGCGCCGAGCTA GGCTTCGGATT 3′
B1-SmBiT-YAP-50-171-F	CCGCGGATCC GATGGTGACCGGCTACCGGCTGT TCGAGGA
N1-Trb-SmBiT-YAP-50-171-R	AAGGAAGCGGCCGCAGATCCACGCGGAACCAGTACATCATCAGGTATCTCA AAAGA
B1- LgBiT-TEAD1-194-411-F	CCGCGGATCC GATGGTCTTCACACTCGAAGATT TCGTTGG
N1-Trb-LgBiT-TEAD1-194-411-R	AAGGAAGCGGCCGCAGATCCACGCGGAACCAGATTTGAAACTTCAAACACA CAGGC

© 2019 by the authors. Licensee MDPI, Basel, Switzerland. This article is an open access article distributed under the terms and conditions of the Creative Commons Attribution (CC BY) license (http://creativecommons.org/licenses/by/4.0/).
